# Impaired coronary flow reserve determined by MR measurement of coronary sinus flow predicts adverse outcome in patients with known or suspected coronary artery disease

**DOI:** 10.1186/1532-429X-15-S1-O91

**Published:** 2013-01-30

**Authors:** Masaki Ishida, Tatsuro Ito, Yasuyuki Shiraishi, Kakuya Kitagawa, Kaoru Dohi, Hiroshi Nakajima, Masaaki Ito, Hajime Sakuma

**Affiliations:** 1 Radiology, Mie University Hospital, Tsu, Japan; 2 Cardiology, Mie University Hospital, Tsu, Japan

## Background

Recent studies demonstrated that ^82^Rb PET-derived myocardial perfusion reserve has a significant prognostic impact on the prediction of cardiac events in patients assessed for myocardial ischemia. Regional myocardial ischemia and late gadolinium enhancement (LGE) defined by cardiac MR imaging are also reported as significant prognosticators for future cardiac events. However, the prognostic value of coronary flow reserve (CFR) obtained by MR flow measurement remains uncertain. The aim of this study was to evaluate the prognostic value of CFR determined by phase contrast cine MR imaging of the coronary sinus in patients with known or suspected coronary artery disease (CAD).

## Methods

203 patients (mean age, 65±13 years; male, 57%) with known or suspected CAD underwent cardiac MR studies including phase contrast cine MRI of coronary sinus, and stress-rest perfusion and LGE MR imaging. The patients with previous revascularization were excluded from this study. Presence or absence of regional ischemia in ≥2 AHA segments and LGE in any myocardial segment were visually determined on stress-rest perfusion and LGE MRI, respectively. CFR was obtained as the ratio of coronary sinus flow assessed by phase contrast cine MRI at baseline and during ATP stress and categorized in tertile (lower, <1.5; middle, 1.5 to 2.0; upper 2.0<). MACEs were defined as cardiac death, late revascularization (>90days), acute myocardial infarction, unstable angina, heart failure, and ventricular arrhythmia.

## Results

During a median follow-up period of 12 months (range, 1 to 28 months), MACEs were observed in 6 (24.0%) of 25 patients with lower CFR tertile, 2 (5.6%) of 36 patients with middle CFR tertile and 6 (4.2%) of 142 patients with upper CFR tertile, corresponding to the annual event rate of 22.7%, 8.2% and 7.2%, respectively. Kaplan-Meier curve demonstrated a significantly lower MACE-free survival rate in patients with lower CFR tertile compared to those with middle and upper CFR tertile (log-rank test, p=0.037 and p=0.003, respectively) (Figure). In a stepwise multivariate Cox model adjusting for age, gender, hypertension, diabetes, hyperlipidemia, smoking, family history of premature CAD, and obesity, the lower CFR tertile (hazard ratio, 3.35; p=0.047), presence of regional ischemia (hazard ratio, 3.60; p=0.032) and presence of LGE (hazard ratio, 9.34; p=0.001) were independent predictors of MACE.

**Figure 1:**
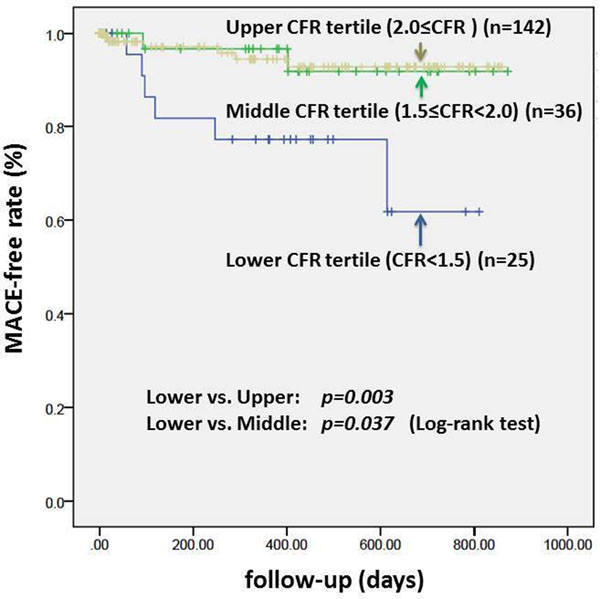
Kaplan-Meier curves showing the difference in MACE-free rate among CFR tertiles (lower, <1.5; middle, 1.5 to 2.0; upper, 2.0<).

## Conclusions

CFR quantified by phase contrast cine MR imaging of the coronary sinus can predicts future major cardiac events in patients with known or suspected CAD independent of the conventional cardiac MR predictors such as presence of regional ischemia and infarction.

## Funding

Departmental research funding.

